# Management of adverse events in patients with acute myeloid leukemia in remission receiving oral azacitidine: experience from the phase 3 randomized QUAZAR AML-001 trial

**DOI:** 10.1186/s13045-021-01142-x

**Published:** 2021-08-28

**Authors:** Farhad Ravandi, Gail J. Roboz, Andrew H. Wei, Hartmut Döhner, Christopher Pocock, Dominik Selleslag, Pau Montesinos, Hamid Sayar, Maurizio Musso, Angela Figuera-Alvarez, Hana Safah, William Tse, Sang Kyun Sohn, Devendra Hiwase, Timothy Chevassut, Francesca Pierdomenico, Ignazia La Torre, Barry Skikne, Rochelle Bailey, Jianhua Zhong, C. L. Beach, Herve Dombret

**Affiliations:** 1 Department of Leukemia, University of Texas MD Anderson Cancer Center, 1515 Holcombe Blvd., Houston, TX 77030 USA; 2 Weill Cornell Medicine, New York, NY USA; 3 New York Presbyterian Hospital, New York, NY USA; 4 Department of Clinical Haematology, The Alfred Hospital, Melbourne, Australia; 5 Australian Centre for Blood Diseases, Monash University, Melbourne, Australia; 6 Department of Internal Medicine III, Ulm University Hospital, Ulm, Germany; 7 Kent & Canterbury Hospital, Canterbury, UK; 8 AZ Sint-Jan Brugge-Oostende AV, Bruges, Belgium; 9 Hospital Universitari i Politècnic La Fe, Valencia, Spain; 10 Indiana University Cancer Center, Indianapolis, IN USA; 11 La Maddalena - Casa di Cura, Palermo, Italy; 12 Hospital Universitario de La Princesa, Madrid, Spain; 13 Tulane University Health Science Center, New Orleans, LA USA; 14 University of Louisville School of Medicine, Louisville, KY USA; 15 Kyungpook National University Hospital, Daegu, Korea; 16 Royal Adelaide Hospital, Adelaide, Australia; 17 Brighton and Sussex Medical School, Brighton, UK; 18 Portuguese Institute of Oncology Lisbon, Lisbon, Portugal; 19 Celgene, a Bristol-Myers Squibb Company, Boudry, Switzerland; 20 University of Kansas Medical Center, Kansas City, KS USA; 21 Bristol Myers Squibb, Princeton, NJ USA; 22 Hôpital Saint-Louis, Assistance Publique – Hôpitaux de Paris (AP-HP), Paris, France; 23 Institut de Recherche Saint-Louis, Université de Paris, Paris, France

**Keywords:** Oral azacitidine, CC-486, Safety, Maintenance

## Abstract

**Background:**

Most older patients with acute myeloid leukemia (AML) who attain morphologic remission with intensive chemotherapy (IC) will eventually relapse and post-relapse prognosis is dismal. In the pivotal QUAZAR AML-001 trial, oral azacitidine maintenance therapy significantly prolonged overall survival by 9.9 months (*P* < 0.001) and relapse-free survival by 5.3 months (*P* < 0.001) compared with placebo in patients with AML in first remission after IC who were not candidates for transplant. Currently, the QUAZAR AML-001 trial provides the most comprehensive safety information associated with oral azacitidine maintenance therapy. Reviewed here are common adverse events (AEs) during oral azacitidine treatment in QUAZAR AML-001, and practical recommendations for AE management based on guidance from international cancer consortiums, regulatory authorities, and the authors’ clinical experience treating patients in the trial.

**Methods:**

QUAZAR AML-001 is an international, placebo-controlled randomized phase 3 study. Patients aged ≥ 55 years with AML and intermediate- or poor-risk cytogenetics at diagnosis, who had attained first complete remission (CR) or CR with incomplete blood count recovery (CRi) within 4 months before study entry, were randomized 1:1 to receive oral azacitidine 300 mg or placebo once-daily for 14 days in repeated 28-day cycles. Safety was assessed in all patients who received ≥ 1 dose of study drug.

**Results:**

A total of 469 patients received oral azacitidine (*n* = 236) or placebo (*n* = 233). Median age was 68 years. Patients received a median of 12 (range 1–80) oral azacitidine treatment cycles or 6 (1–73) placebo cycles. Gastrointestinal AEs were common and typically low-grade. The most frequent grade 3–4 AEs during oral azacitidine therapy were hematologic events. AEs infrequently required permanent discontinuation of oral azacitidine (13%), suggesting they were effectively managed with use of concomitant medications and oral azacitidine dosing modifications.

**Conclusion:**

Oral azacitidine maintenance had a generally favorable safety profile. Prophylaxis with antiemetic agents, and blood count monitoring every other week, are recommended for at least the first 2 oral azacitidine treatment cycles, and as needed thereafter. Awareness of the type, onset, and duration of common AEs, and implementation of effective AE management, may maximize treatment adherence and optimize the survival benefits of oral azacitidine AML remission maintenance therapy.

*Trial registration* This trial is registered on clinicaltrials.gov: NCT01757535 as of December 2012.

**Supplementary Information:**

The online version contains supplementary material available at 10.1186/s13045-021-01142-x.

## Background

Acute myeloid leukemia (AML) is primarily a disease of older individuals; median age at diagnosis is 68 years [1]. About 40–60% of patients aged ≥ 60 years with AML attain morphologic remission (< 5% blasts in bone marrow [2]) with intensive chemotherapy (IC), but most of these patients will eventually relapse [3, 4]. Effective, well-tolerated maintenance therapies are needed to prolong remission, reduce risk of relapse, and improve survival for older patients with AML. Maintenance therapy for AML has been controversial historically due to limited efficacy and the potential for substantial treatment-related toxicity [5–7]. Additionally, patients with AML in remission generally report improving health-related quality of life (HRQoL) over time [8] and may be ambivalent toward receiving further active therapy that can induce unwanted effects [9].

Azacitidine is a DNA methyltransferase inhibitor and hypomethylating agent (HMA) that, when administered parenterally for 5–7 days per 28-day treatment cycle, induces clinical responses and prolongs overall survival (OS) as front-line treatment in older patients with AML or higher-risk myelodysplastic syndromes (MDS) [10, 11]. Injectable azacitidine has been shown to prolong disease-free survival (DFS)—but not OS—as maintenance therapy in patients ≥ 60 years of age with AML in remission after IC [12]. Oral azacitidine (formerly CC-486) has been evaluated in patients with hematologic malignancies in multiple clinical trials [13–19]. Oral administration allows for extended dosing schedules (> 7 days per cycle) to sustain the therapeutic activity of azacitidine across the entire treatment cycle. Importantly, the oral and parenteral formulations of azacitidine are not bioequivalent and not interchangeable [20].

**Fig. 1:**
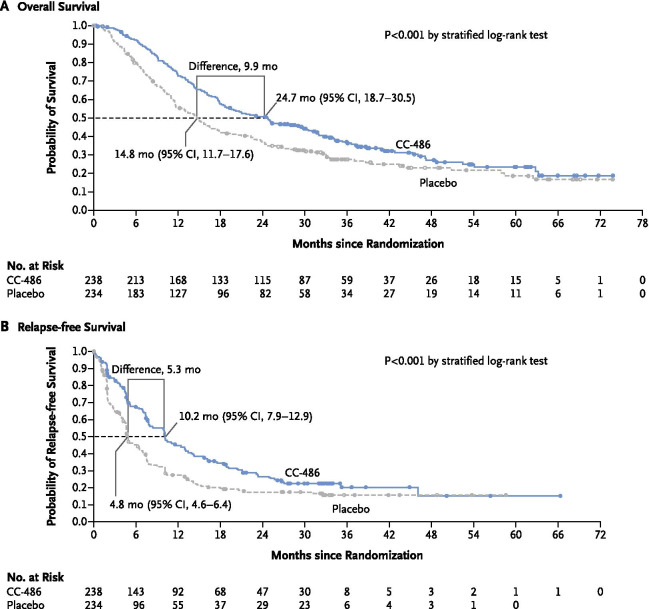
Kaplan–Meier analysis of overall survival and relapse-free survival from the time of randomization. From Wei et al. [22]. Copyright © (2021) Massachusetts Medical Society. Reprinted with permission

Oral azacitidine is approved in the United States, European Union, and Canada for treatment of adult patients with AML in first remission after IC who are not eligible to complete intensive curative therapy (e.g., hematopoietic stem cell transplant [HSCT]) [20, 21]. These regulatory approvals were based on results of the pivotal randomized, phase 3 QUAZAR AML-001 trial, which assessed oral azacitidine as maintenance therapy for patients aged ≥ 55 years with AML in first remission after IC (induction ± consolidation) who were not candidates for HSCT [22]. In that trial, treatment with oral azacitidine 300-mg once-daily 14 days per 28-day cycle was associated with significantly prolonged OS (median 24.7 vs. 14.8 months from randomization; *P* < 0.001) and relapse-free survival (RFS) (median 10.2 vs. 4.8 months; *P* < 0.001) compared with placebo (Fig. 1) [22]. Hematologic and gastrointestinal (GI) adverse events (AEs) were common in both treatment arms, but rates of these events were higher with active treatment.

GI events are among the most common AEs to occur during treatment with both formulations of azacitidine; these are typically low-grade and occur most often during early treatment cycles [19, 23]. Because cytotoxic chemotherapies act on healthy myeloproliferative cells in addition to leukemic targets, many AML treatments, including HMAs, have myelosuppressive effects that may induce or exacerbate cytopenias [24]. These AEs could negatively affect patients’ HRQoL and interfere with successful maintenance therapy, potentially leading to therapeutic nonadherence or treatment discontinuation [25], so it is important to effectively manage common AEs during oral azacitidine therapy.

Reported here are rates of common GI and hematologic AEs and infections during oral azacitidine treatment in the QUAZAR AML-001 trial, and associated management strategies, supportive care guidelines from international experts who care for patients with AML [26–33], and suggestions based on the authors’ clinical experience treating patients with oral azacitidine in QUAZAR AML-001.

## Methods

### QUAZAR AML-001 overview

Comprehensive study design, patient eligibility, and overall efficacy and safety outcomes for QUAZAR AML-001 (NCT01757535) are reported elsewhere [22]. Briefly, eligible patients were aged ≥ 55 years with newly diagnosed AML in first complete remission (CR) or CR with incomplete blood count recovery (CRi) after induction chemotherapy, with or without subsequent consolidation courses, and had intermediate- or poor-risk cytogenetics, Eastern Cooperative Oncology Group performance status (ECOG PS) score of ≤ 2, absolute neutrophil count (ANC) ≥ 0.5 × 10^9^/L, and platelet count ≥ 20 × 10^9^/L at screening. Within 4 months of achieving CR/CRi, patients were randomized 1:1 to receive oral azacitidine 300-mg or placebo once-daily for 14 days of repeated 28-day treatment cycles. All patients could receive supportive care measures according to local practice, including (but not limited to) red blood cell (RBC) transfusions, platelet transfusions, and erythropoiesis stimulating agents (ESAs); antibiotic, antiviral, and antifungal therapies; and myeloid growth factors (e.g., granulocyte colony stimulating factor [G-CSF]).

Safety was assessed in all patients who received ≥ 1 dose of study drug, per investigator reporting of treatment-emergent AEs occurring between the date of first study drug dose through 28 days after last dose. AEs were defined using the Medical Dictionary for Regulatory Activities (MedDRA) version 22.0 and graded for severity using the National Cancer Institute Common Terminology Criteria for Adverse Events (NCI-CTCAE) version 4.0. Any AE that resulted in death, was life-threatening, required or prolonged inpatient hospitalization, resulted in significant disability, or led to congenital anomalies or other important medical events was considered a serious event. Study drug dosing could be interrupted, delayed, or reduced to manage AEs. While development of ≥ 5% bone marrow blasts is considered morphologic AML relapse [2], patients who experienced AML relapse with 5–15% blasts in blood or bone marrow could have their randomized dosing regimen extended from 14 to 21 days/cycle at the discretion of the treating physician. Treatment discontinuation was mandatory for patients with > 15% blasts. This report excludes MedDRA terms specifically related to AML relapse (e.g., high-level term “leukemia”) because relapse was an efficacy measure of the trial.

## Results

### Patients and overall safety in QUAZAR AML-001

In all, 472 patients were randomized to treatment; the safety-evaluable population comprised 469 patients (99%) who received ≥ 1 dose of oral azacitidine (*n* = 236) or placebo (*n* = 233). Median age overall was 68 years (range 55–86). In the oral azacitidine arm, 86% of patients had intermediate-risk cytogenetics at AML diagnosis, 78% had achieved CR after induction, and 78% received ≥ 1 course of consolidation before study randomization. Median baseline ANC was 3.0 × 10^9^/L, hemoglobin concentration was 11.3 g/dL, platelet count was 154 × 10^9^/L, and white blood cell (WBC) count was 4.9 × 10^9^/L (Table 1).

**Table 1:** QUAZAR AML-001 baseline characteristics (safety-evaluable population)

Parameter	Oral azacitidine (*N* = 236)	Placebo (*N* = 233)
Age, years, median (range)	68 (55‒86)	68 (55‒82)
*ECOG PS score, n (%)*		
0	116 (49.2)	111 (47.6)
1	99 (41.9)	105 (45.1)
2‒3	21 (8.9)	17 (7.3)
De novo AML, *n* (%)	211 (89.4)	215 (92.3)
*WHO AML classification, n (%)*		
Not otherwise specified	147 (62.3)	144 (61.8)
Myelodysplasia-related changes	49 (20.8)	42 (18.0)
Recurrent genetic abnormalities	38 (16.1)	46 (19.7)
Therapy-related	2 (0.8)	0
*NCCN cytogenetic risk at diagnosis, n (%)*		
Intermediate	202 (85.6)	202 (86.7)
Poor	34 (14.4)	31 (13.3)
*Response after induction, n (%)*		
CR	185 (78.4)	197 (84.5)
Cri	51 (21.6)	36 (15.5)
*Received consolidation, n (%)*		
Yes	184 (78.0)	191 (82.0)
1 cycle	110 (46.6)	101 (43.3)
2 cycles	69 (29.2)	77 (33.0)
3 cycles	5 (2.1)	13 (5.6)
No	52 (22.0)	42 (18.0)
*Reason ineligible for transplant*^*a*^		
Age	154 (65)	152 (65)
Comorbidities	52 (22)	50 (21)
Performance status	14 (6)	9 (4)
No available donor	37 (16)	35 (15)
Patient decision	19 (8)	32 (14)
Unfavorable cytogenetics	6 (3)	10 (4)
Other	28 (12)	21 (9)
Time from induction to randomization, months, median (range)	3.9 (1.4‒8.8)	4.0 (1.3‒15.1)
Time from CR/CRi to randomization, days, median (range)	84 (7‒154^b^)	86 (7‒263^b^)
Bone marrow blasts, percent, median (range)	2.0 (0.0‒5.0)	2.0 (0.0‒6.5^c^)
MRD + at randomization,^d^ *n* (%)	102 (43.2)	115 (49.4)
ANC, 10^9^/L, median (range)	3.0 (0.3^b^‒15.9)	2.8 (0.5‒9.6)
Hemoglobin, g/dL, median (range)	11.3 (7.5‒15.9)	10.8 (7.7‒14.9)
Platelets, 10^9^/L, median (range)	154 (22‒801)	179 (16^b^‒636)
WBC, 10^9^/L, median (range)	4.9 (0.8‒18.7)	4.5 (1.3‒12.6)

Safety-evaluable patients received at least 1 dose of study drug

*AML* acute myeloid leukemia, *ANC* absolute neutrophil count, *CR* complete remission, *CRi* CR with incomplete blood count recovery, *ECOG PS* Eastern Cooperative Oncology Group performance status, *MRD* measurable residual disease, *NCCN* National Comprehensive Cancer Network, *WBC* white blood cells, *WHO* World Health Organization

^a^A patient may have had more than 1 reason for ineligibility

^b^Two patients in each treatment arm enrolled beyond the 4-month (± 7 days) inclusion window from the time of achieving CR/CRi (protocol violations)

^c^Patients may have had multiple visits between screening and randomization. All patients met relevant eligibility criteria at the screening visit

^d^Central assessment by flow cytometry, using a ≥ 0.1% MRD-positive threshold

Oral azacitidine had a manageable safety profile overall, with no unexpected safety events. Patients received a median of 12 (range 1–80) oral azacitidine treatment cycles or 6 (1–73) cycles of placebo [22]. In both treatment arms, the median cycle length was 28 days, median number of days treated per cycle was 14, and median compliance rate was 100%. AEs led to temporary dosing interruptions for 43% of patients who received oral azacitidine and 17% of patients in the placebo arm, and required dose reductions for 16% and 3% of patients, respectively. AEs infrequently led to permanent discontinuation of oral azacitidine (*n* = 31; 13%) or placebo (*n* = 10; 4%) [22].

The most common AEs in both treatment arms were grade 1–2 GI events (Table 2). The most frequent grade 3–4 AEs (reported as events or indicated by laboratory measures) in both treatment arms were cytopenias, primarily neutropenia (in 41% of patients in the oral azacitidine arm and 24% in the placebo arm), thrombocytopenia (in 22% and 21%, respectively), and anemia (in 14% and 13%) [22]. Serious AEs reported in ≥ 1% of patients are shown in Additional file 1: Table S1. Hospitalization (for any cause, including AML relapse) was required for 46% of patients in the oral azacitidine arm and 51% of patients in the placebo arm. When accounting for different durations of exposure to study drugs, exposure-adjusted incidence rates of hospitalization and days in hospital were each significantly lower during oral azacitidine treatment compared with placebo [34].

**Table 2:** Most common (≥ 20%) adverse events (all grades and grade 3–4) in patients treated with oral azacitidine in QUAZAR AML-001 [22]

	Oral azacitidine (*N* = 236)		Placebo (*N* = 233)	
	All grades	Grade 3–4	All grades	Grade 3–4
	*n* (%)			
Patients with ≥ 1 AE	231 (98)	169 (72)	225 (97)	147 (63)
Nausea	153 (65)	6 (3)	55 (24)	1 (< 1)
Vomiting	141 (60)	7 (3)	23 (10)	0
Diarrhea	119 (50)	12 (5)	50 (21)	3 (1)
Neutropenia	105 (44)	97 (41)	61 (26)	55 (24)
Constipation	91 (39)	3 (1)	56 (24)	0
Thrombocytopenia	79 (33)	53 (22)	63 (27)	50 (21)
Fatigue	70 (30)	7 (3)	45 (19)	2 (1)
Anemia	48 (20)	33 (14)	42 (18)	30 (13)

Adverse events were evaluated from the date of the first dose of oral azacitidine or placebo through 28 days after the last dose. Preferred terms were defined using the Medical Dictionary of Regulatory Activities, version 22.0, and events were graded using the National Cancer Institute Common Terminology Criteria for Adverse Events, version 4.0. Patients are counted only once for multiple events within each preferred term

*AE* adverse event

### Gastrointestinal events

**Fig. 2:**
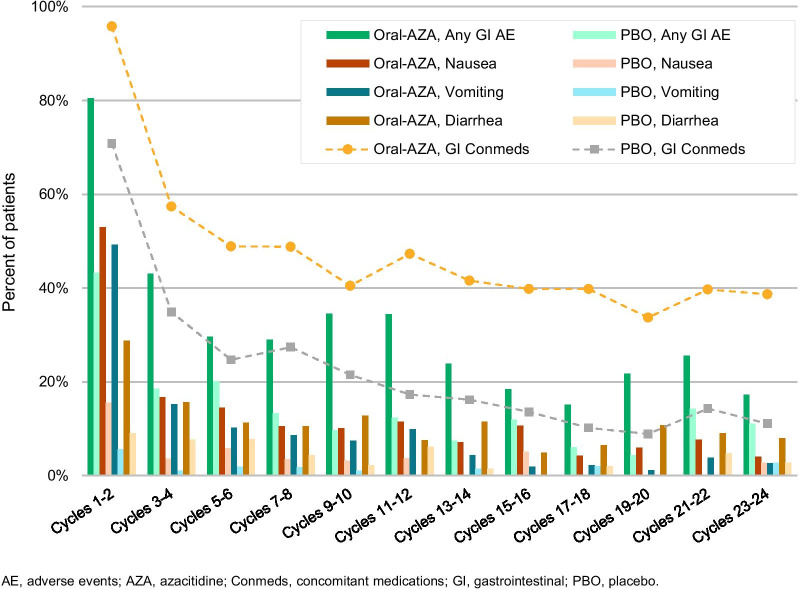
Rates of all gastrointestinal adverse events; of nausea, vomiting, and diarrhea; and use of gastrointestinal-directed concomitant medications over time in the QUAZAR AML-001 trial

GI-directed prophylaxis was allowed but not mandatory in QUAZAR AML-001; the study protocol included dose-modification recommendations for grade ≥ 3 GI AEs (diarrhea, nausea, and vomiting). Overall, GI AEs occurred in 91% and 62% of patients in the oral azacitidine and placebo arms, respectively. The most common GI events in the oral azacitidine arm were nausea (65%), vomiting (60%), and diarrhea (50%), and most GI events were low-grade (Table 3). Few patients in the oral azacitidine arm experienced grade-3 nausea (3%), vomiting (3%), or diarrhea (5%), and only 1 of these events was grade 4 in severity (diarrhea) at any time on-study. Serious GI events were reported for 15 patients (6%) in the oral azacitidine arm; the only serious events reported for > 1 patient were diarrhea (*n* = 3), gastritis (*n* = 2), and vomiting (*n* = 2). GI events occurred most frequently during the first 2 treatment cycles and decreased in incidence thereafter. In cycles 1–2, 3–4, and 5–6, respectively, nausea was reported in 53%, 17%, and 15% of patients receiving oral azacitidine; vomiting occurred in 49%, 15%, and 10% of patients; and diarrhea occurred in 29%, 16%, and 11% of patients (Fig. 2).

**Table 3:** Maximum severity of gastrointestinal adverse events reported in > 5% of patients receiving oral azacitidine

Preferred term	Total	Oral azacitidine (*N* = 236)				
		Maximum CTCAE grade				
		1	2	3	4	5
		*n* (%)				
Any GI adverse event	215 (91)	63 (27)	118 (50)	30 (13)	4 (2)	0
Nausea	153 (65)	81 (34)	66 (28)	6 (3)	0	0
Vomiting	141 (60)	81 (34)	53 (22)	7 (3)	0	0
Diarrhea	119 (50)	62 (26)	45 (19)	11 (5)	1 (< 1)	0
Constipation	91 (39)	52 (22)	36 (15)	3 (1)	0	0
Abdominal pain	31 (13)	19 (8)	10 (4)	2 (1)	0	0
Upper abdominal pain	21 (9)	14 (6)	5 (2)	2 (1)	0	0
Flatulence	13 (6)	9 (4)	4 (2)	0	0	0

Adverse events were evaluated from the date of the first dose of oral azacitidine or placebo through 28 days after the last dose. Preferred terms were defined using the Medical Dictionary of Regulatory Activities, version 22.0, and events were graded using the National Cancer Institute Common Terminology Criteria for Adverse Events, version 4.0. Patients are counted only once for multiple events within each preferred term

*CTCAE* Common Terminology Criteria for Adverse Events, *GI* gastrointestinal

#### Management

GI AEs were primarily managed with standard supportive care measures and oral azacitidine dosing modifications. Modifications to oral azacitidine dosing for nausea, vomiting, and diarrhea were mainly treatment interruptions (for 6%, 4%, and 4% of patients, respectively) and dose reductions (3%, 2%, and 1%), and these events rarely required treatment discontinuation (2%, 2%, and 1%). The use of concomitant medications for nausea, vomiting, and diarrhea decreased over time in parallel with rates of GI AEs as therapy continued (Fig. 2). The most common GI-directed concomitant medications in the oral azacitidine arm were 5-HT3 receptor antagonists [ondansetron (83%), metoclopramide (34%), granisetron (18%)], and proton pump inhibitors [PPIs; pantoprazole (30%), omeprazole/esomeprazole (29%)]. Intravenous fluids (e.g., NaCl, electrolyte solutions, mannitol) were administered for approximately 10% of patients in each arm. Median durations of nausea, vomiting, and diarrhea were 10 days, 2 days, and 6 days, respectively. As mentioned, antiemetic prophylaxis was not mandated during the trial.

**Fig. 3:**
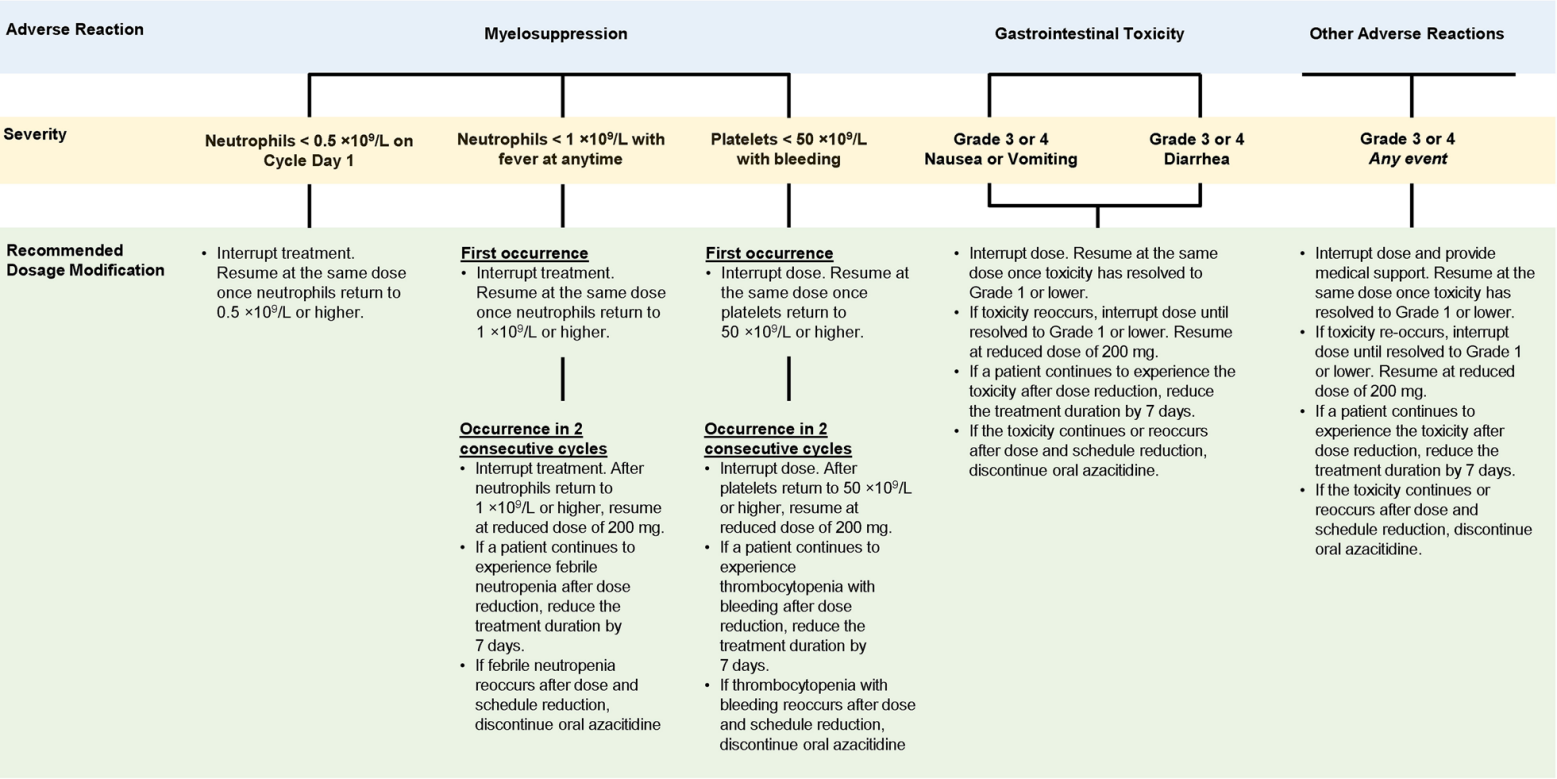
Recommended dosing modifications for adverse events during oral azacitidine therapy

Oral azacitidine can be taken with or without food [35, 36]. At clinically relevant concentrations, oral azacitidine does not substantially inhibit or induce cytochrome P450 enzymes [20], decreasing the risk of adverse drug interactions, and oral azacitidine pharmacokinetics are not meaningfully altered by coadministration of a PPI [36]. Treatment of GI reflux with a PPI or histamine H2-receptor antagonist (e.g., cimetidine, famotidine) may ameliorate dyspepsia, which can mimic nausea [37]. Taking oral azacitidine with food may alleviate nausea and vomiting by diluting drug in the upper GI tract, improving motility, and slowing the rate of absorption [25]. The National Comprehensive Care Network (NCCN) guidelines for antiemesis recommend prophylactic use of a 5-HT3 receptor antagonist before the start of moderately or highly emetogenic therapy [37]. Accordingly, the prescribing information for oral azacitidine recommends use of anti-nausea prophylaxis before taking oral azacitidine for at least the first 2 treatment cycles [20]. Although higher-grade GI events are uncommon, oral azacitidine treatment should be interrupted for patients who experience grade 3–4 nausea, vomiting, or diarrhea, with treatment resumed once toxicity resolves to grade ≤ 1. If grade 3–4 events reoccur, treatment should again be interrupted until resolution to grade ≤ 1; treatment should be resumed at a lower dose (200 mg/day) on second incidence, followed by a shorter treatment duration (reduced by 7 days) if the toxicity persists after dose reduction (Fig. 3) [20].

Consensus clinical practice guidelines emphasize use of 3 drug classes for optimal treatment of chemotherapy-induced nausea and vomiting: 5-HT3 receptor antagonists, corticosteroids, and neurokinin-1 receptor antagonists [28, 33, 37]. Nonpharmacologic approaches to reduce nausea and vomiting are shown in Additional file 1: Table S2. Uncomplicated diarrhea often can be managed by replacement of fluids and electrolytes and self-administration of antidiarrheal medications (e.g., loperamide, lactulose, bismuth subsalicylate). If diarrhea is grade ≥ 3 in severity, oral azacitidine dosing should be interrupted until it resolves to grade ≤ 1, then resumed at the recommended starting dose—unless there is a reoccurrence, in which case a reduced dose (200 mg/day) and/or a reduced treatment duration (e.g., 7 days/cycle) should be considered.

### Hematologic events

Most treatments for hematologic malignancies have myelosuppressive effects [23, 38, 39]. In the QUAZAR AML-001 trial, hematologic AEs were reported in 66% of patients (155/236) in the oral azacitidine arm and 47% (110/233) in the placebo arm. The most common hematologic AEs were neutropenia, thrombocytopenia, and anemia, which were reported for 44%, 33%, and 20% of patients in the oral azacitidine arm, respectively, and in 26%, 27%, and 18% of patients in the placebo arm [22]. Some hematologic AEs may have been subclinical, detected only because of protocol-required frequent laboratory monitoring during the study; for example, while neutropenia was reported in 44% of patients receiving oral azacitidine, febrile neutropenia occurred in only 12% of these patients (and in 8% of patients in the placebo arm), and thrombocytopenia was reported in 33% of patients receiving oral azacitidine but hemorrhagic events were reported for 22% of patients (and in 20% of patients receiving placebo). Frequent monitoring also allowed for prompt detection of severe neutropenia or thrombocytopenia, and the need for oral azacitidine dosing modifications.

**Fig. 4:**
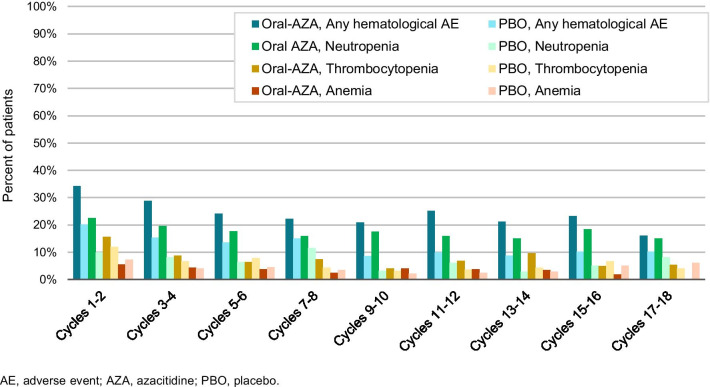
Rates of all hematologic adverse events, and of neutropenia, thrombocytopenia, and anemia over time in the QUAZAR AML-001 trial

The frequency of hematologic AEs during initial treatment cycles was relatively consistent in the oral azacitidine arm, with a downward trend in later cycles (Fig. 4). During cycles 1–2, 3–4, and 5–6, rates of neutropenia in the oral azacitidine arm were 22%, 20%, and 18%, respectively; of thrombocytopenia were 16%, 9%, and 6%; and of anemia were 6%, 4%, and 4%. Grade 3–4 neutropenia, thrombocytopenia, and anemia occurred in 41%, 22%, and 14% of patients, respectively, in the oral azacitidine arm. Serious febrile neutropenia was reported for 7% of patients receiving oral azacitidine (placebo 4%).

**Fig. 5:**
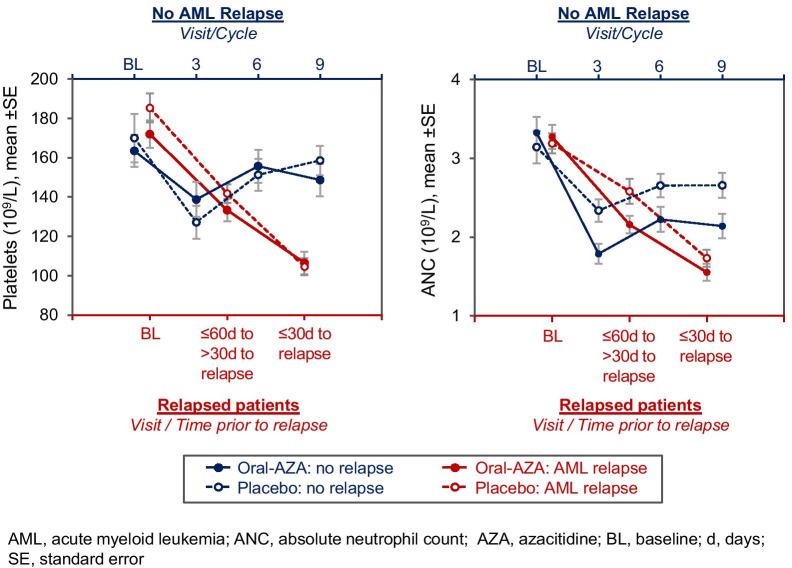
Changes in platelet and neutrophil counts in the lead-up to AML relapse (and at cycles 3, 6, and 9 for patients who did not relapse) in the QUAZAR AML-001 trial

While cytopenias are a known side effect of HMAs, they may also accompany AML relapse, and it can be challenging to determine whether cytopenias during oral azacitidine therapy are drug-related or reflect disease progression. In QUAZAR AML-001, 154 patients (65%) in the oral azacitidine arm and 179 (77%) in the placebo arm relapsed on-study. As shown in Fig. 4, rates of hematologic AEs decreased slightly over time but generally ranged from 15 to 30% in the oral azacitidine arm and 8–15% in the placebo arm from cycle 3 through cycle 18, and it remains unclear how many of these events were potentially related to onset of AML relapse. In a post hoc analysis, ANC, platelet counts, and hemoglobin concentrations were assessed at baseline, 30–60 days before the date of recorded relapse, and within 30 days of relapse (inclusive of the relapse date) for the 333 patients who relapsed during the QUAZAR AML-001 study. For comparison purposes, these parameters were also assessed at baseline and at cycles 3, 6, and 9 for non-relapsing patients. Results showed steep decreases in ANC and platelets (Fig. 5) but not hemoglobin, in the lead-up to relapse for patients in both treatment arms. In contrast, patients who did not relapse showed initial decreases in platelet counts and ANC, but signs of count recovery were evident after cycle 3.

#### Management

Clinicians should monitor blood counts during oral azacitidine treatment every-other week for the first 2 cycles and before the start of each cycle thereafter [20]. More frequent monitoring is also recommended for the 2 treatment cycles following any dose reduction for myelosuppression [20].

Hematologic AEs in the oral azacitidine arm in QUAZAR AML-001 were primarily managed with dosing modifications, including treatment interruption in 27% and dose reductions in 8% of patients. Only 3 patients discontinued oral azacitidine due to a hematologic AE. Oral azacitidine dosing should be delayed for patients with ANC < 0.5 × 10^9^/L at the beginning of any treatment cycle [20]. For patients who develop febrile neutropenia (ANC < 1 × 10^9^/L with fever) or thrombocytopenia (platelets < 50 × 10^9^/L) during oral azacitidine treatment, treatment should be interrupted until count recovery (ANC ≥ 1 × 10^9^/L, platelets ≥ 50 × 10^9^/L). If multiple events of neutropenic fever or thrombocytopenia occur in consecutive cycles, oral azacitidine should again be interrupted until recovery and resumed at a lower dose and/or reduced schedule (Fig. 3). As part of prospective study design of the QUAZAR AML-001 trial, patients who experienced febrile neutropenia could continue receiving study drug—along with antibiotic, antifungal, or antimicrobial agents—for up to 3 days before treatment interruption and study drug could be resumed no earlier than 3 days following fever resolution.

Among the 105 patients who experienced neutropenia during oral azacitidine treatment, 51 had oral azacitidine dosing interrupted [*n* = 47 (45%)] and/or reduced [*n* = 13 (12%)], typically due to grade 3–4 events (*n* = 49/51). Myeloid growth factors, including G-CSF/filgrastim, were used in 50 patients (21%) in the oral azacitidine arm and in 36 patients (15%) in the placebo arm. Intermittent G-CSF treatment may be helpful for patients with grade-4 neutropenia during oral azacitidine treatment. Expert guidelines recommend prophylactic use of G-CSF when the overall risk of febrile neutropenia from a chemotherapy regimen is ≥ 20% of patients [27, 40]; the frequency of febrile neutropenia during oral azacitidine treatment did not meet this threshold (12%).

During oral azacitidine treatment, 23 of 79 patients who experienced thrombocytopenia had dosing interrupted [*n* = 20 (25%)] and/or reduced (*n* = 4 [5%]), including 15 patients who had their dosing modified because of grade 3–4 events. Platelet transfusions were administered for 19% of patients receiving oral azacitidine and 22% receiving placebo. No patient in the oral azacitidine arm received fibrinogen and only 1 patient received prothrombin on-study. NCCN clinical practice guidelines recommend dose modifications, platelet transfusions, and use of romiplostim for patients with substantial chemotherapy-induced thrombocytopenia [31].

Supportive care measures for 48 patients in the oral azacitidine arm who experienced anemia included treatment interruptions for 3 patients (6%) and dose reduction for 1 patient (2%). Rates of RBC transfusions on-study were similar between the oral azacitidine and placebo arms, in 23% and 22% of patients, respectively, and only 1 patient received an ESA (epoetin alpha). Guidelines from the American Society of Clinical Oncology (ASCO) and the American Society of Hematology (ASH) indicate that ESAs may benefit patients with hemoglobin levels ≤ 10 g/dL [41], and NCCN clinical practice guidelines for chemotherapy-induced anemia recommend ESAs for symptomatic patients with hemoglobin ≤ 11 g/dL [31].

### Infections

AEs in the MedDRA System Organ Class “Infections and infestations” were reported for 62% of patients treated with oral azacitidine and 53% of patients in the placebo arm. Most infectious events were not considered study-drug-related by investigators; 12% and 3% in the oral azacitidine and placebo arms, respectively, were considered treatment-related. The most common infections in the oral azacitidine arm were upper respiratory tract infections in 13% of patients, influenza in 8%, nasopharyngitis in 7% and urinary tract infections in 7%; rates of these events in the placebo arm were 14%, 3%, 7%, and 6%, respectively. The prescribing information for oral azacitidine reports an incidence of pneumonia of 27% (including serious pneumonia in 8% of patients), but in that document, “pneumonia” encompasses a broad range of disorders in addition to different types of pneumonia, including influenza, respiratory tract infection, bronchopulmonary aspergillosis, lung infection, productive cough, and pleural effusion, among others [20]. AEs with the preferred term of “pneumonia” were reported in 6% of patients in the oral azacitidine arm and 5% of patients in the placebo arm, with grade 3–4 events reported for 7 patients (3%) in each treatment arm.

Infections were the most frequent serious AEs in both the oral azacitidine (17%) and placebo (8%) arms. Serious infections in > 1 patient receiving oral azacitidine were pneumonia (4%), cellulitis (2%), sepsis (2%), and influenza (1%); these events were reported in 3% 0.4%, 2%, and 0% of patients in the placebo arm, respectively. Serious AEs were considered related to oral azacitidine treatment in 11 patients (5%), with pneumonia (*n* = 6; 3%) and sepsis (*n* = 2; 1%) reported in > 1 patient. The median duration of serious pneumonia in the oral azacitidine arm (9 events) was 12 days, of sepsis (4 events) was 16 days, and of cellulitis (4 events) was 16 days. In the placebo arm, median durations of serious pneumonia and sepsis (no cellulitis) were similar to the oral azacitidine arm (14 days and 16 days, respectively).

Three patients who received oral azacitidine had infections that ultimately led to death. Each of these events occurred in the context of AML relapse and after cessation of oral azacitidine treatment, and all 3 patients had received subsequent chemotherapy on or before the date of the infection leading to death.

#### Management

Overall, 13% of patients in QUAZAR AML-001 had oral azacitidine treatment interrupted and 1% required dose reductions due to infections. Treatment was discontinued for 4 patients (2%) due to sepsis, pneumonia, rectal abscess, or lung abscess; sepsis was considered by the investigator to be related to oral azacitidine. The most common concomitant antibiotics, anti-infectives, and antifungal treatments during the trial were ciprofloxacin (22% of patients), levofloxacin (22%), and acyclovir (20%), which were used with similar frequencies in the placebo arm (16%, 19%, and 22%, respectively).

Prophylactic use of G-CSF can reduce the incidence of infection-related mortality in patients with cancer [27]. Additionally, ASCO and Infectious Diseases Society of America (IDSA) guidelines recommend antibacterial and antifungal prophylaxis for patients who are at high risk of infection, including patients who may be expected to have profound, protracted neutropenia (< 1.0 × 10^9^/L neutrophils for > 7 days) [42].

## Discussion

An ideal maintenance therapy should not only prevent or delay relapse and prolong OS, but should also have a manageable safety profile that is conducive to long-term use. Hematologic and GI events were the most common AEs reported in patients who received oral azacitidine in the QUAZAR AML-001 trial; these events were generally manageable with dose interruptions or reductions, and discontinuation of oral azacitidine because of AEs was infrequent. Most GI AEs were low-grade, and frequencies of these events decreased after initial treatment cycles. Because the study was blinded, use of antiemetics during early treatment cycles may have been infrequent, administered on a “wait and see” basis. GI events and use of concomitant GI medications decreased concurrently, suggesting progressive GI tolerance to oral azacitidine. Patient education and behavioral support can improve medication adherence [43], so clinicians and patients should be aware of the likelihood of GI AEs during early oral azacitidine treatment. Prophylaxis with antiemetics during at least the first 2 treatment cycles, and as-needed symptomatic intervention, may facilitate treatment compliance.

Hematologic AEs were also relatively common during oral azacitidine treatment. Neutropenia, thrombocytopenia, and anemia were primarily managed with temporary treatment delays or interruptions and modifications to oral azacitidine dose level. Approximately one-fifth of patients (21%) in the oral azacitidine arm received a myeloid growth factor on-study. It may be prudent to consider prophylaxis for neutropenia during early oral azacitidine treatment for patients with low pretreatment ANC. In many cases, hematologic AEs in this trial were subclinical and not associated with cytopenic complications, detected only because of the required frequent laboratory monitoring. Some hematologic AEs in the oral azacitidine arm may have been associated with AML relapse rather than treatment-related myelosuppression; post hoc analysis of data from QUAZAR AML-001 suggests that patients with declining ANC and platelet counts that do not recover after the first few treatment cycles may be headed toward relapse. Similar trends for reduced platelet and neutrophil counts within 60 days of relapse in the placebo arm (Fig. 5) also suggest these cytopenias were not entirely related to oral azacitidine therapy.

International consensus guidelines for supportive care strategies to address frequent AEs associated with cancer treatment have been updated, in part, to minimize hospital or clinic visits to avoid exposure to nosocomial infection [27]. Reducing the need for hospital or clinic visits is more convenient for patients and reduces healthcare resource utilization. Compared with injectable azacitidine, there is no need for repeated daily clinic visits each month for oral azacitidine administration. Moreover, in a prospective analysis of hospitalization and healthcare resource utilization during the QUAZAR AML-001 study, oral azacitidine was associated with significantly reduced exposure-adjusted rates of hospitalization and days in hospital compared with placebo [34]. Infections were the most common serious AEs in both treatment arms in QUAZAR AML-001, but did not frequently require dosing modifications or discontinuation.

Maintenance therapy has historically not been a component of standard AML care because the toxicity of the regimens under study sometimes outweighed their benefits [5–7]. Therefore, it is especially important to consider effects of maintenance therapy on the HRQoL of older patients with AML in remission, as these effects may determine whether a patients chooses to receive it. HRQoL was a secondary endpoint of the QUAZAR AML-001 trial. Patients in both treatment arms reported levels of fatigue and overall HRQoL at study entry that were similar to those of a general population [44]. The relatively favorable levels of fatigue and overall HRQoL at study entry were preserved during oral azacitidine treatment, with no clinically meaningful differences in patient-reported HRQoL scores from baseline, or from scores in the placebo arm at any post-baseline visit [44].

Because oral azacitidine received regulatory approvals only recently, QUAZAR AML-001 currently comprises the most robust dataset for assessing its safety in patients with AML in remission. Patients in the QUAZAR AML-001 trial may not be typical of a “general” older AML population, as they were eligible for and responded to IC, and were deemed healthy enough to participate in a clinical trial (but not considered fit enough for transplant in most cases). Additional safety information for patients treated with oral azacitidine outside of a clinical trial is anticipated.

Patients prefer oral agents, as long as efficacy is not compromised [45]; oral azacitidine significantly improved OS and RFS without diminishing HRQoL, and is convenient for use in the outpatient setting. Nevertheless, a concern of oral anticancer agents is ensuring drug adherence, which largely depends on patients’ perceptions of risk/benefit/cost (including toxicity) and the complexity of the treatment regimen, to maximize therapeutic efficacy [45]. Awareness of the onset and duration of common AEs, and implementation of effective supportive care for AE management may maximize treatment adherence and optimize the survival benefits of oral azacitidine maintenance therapy for patients with AML in remission.

## Conclusions

Patients who attain AML remission with IC generally begin to feel better, and despite the potential to obtain survival benefits with maintenance therapy, they may not be willing to continue receiving active treatment, especially if the drug regimen confers significant or unexpected toxicity. It is reassuring that the generally favorable HRQoL associated with AML remission at entry to the QUAZAR AML-001 study was not diminished during oral azacitidine therapy. Oral azacitidine maintenance had a manageable safety profile, with no unexpected safety events in patients receiving it long-term (> 5 years). To promote adherence to therapy, clinicians should consider prophylactic use of antiemetics and symptomatic interventions during early oral azacitidine treatment to ameliorate GI events. Hematologic AEs during oral azacitidine therapy rarely required treatment discontinuation and were mainly managed by treatment delays, interruptions, or oral azacitidine dosing modifications. Discerning treatment-related hematologic AEs from cytopenias related to relapse can be challenging; neutropenia or thrombocytopenia that does not show signs of improving may signal impending relapse, but further investigation is needed to confirm this finding. Broader access to a wide range of patients with AML in the “real world” should further improve our understanding of the safety of oral azacitidine maintenance therapy.

## Supplementary Information

**Additional file 1.** Additional file of Management of adverse events in patients with acute myeloid leukemia in remission receiving oral azacitidine: experience from the phase 3 randomized QUAZAR AML-001 trial.

## Data Availability

BMS policy on data sharing may be found at https://www.bms.com/researchers-and-partners/independent-research/data-sharing-request-process.html.

## Abbreviations

AML: Acute myeloid leukemia
ANC: Absolute neutrophil count
AE: Adverse event
ASCO: American Society of Clinical Oncology
ASH: American Society of Hematology
CR: Complete remission
CRi: CR with incomplete blood count recovery
DFS: Disease-free survival
ECOG PS: Eastern Cooperative Oncology Group performance status
ESA: Erythropoiesis stimulating agent
FACIT: Functional Assessment of Chronic Illness Therapy
GI: Gastrointestinal
G-CSF: Granulocyte colony stimulating factors
HMA: Hypomethylating agent
HRQoL: Health-related quality of life
HSCT: Hematopoietic stem cell transplant
IDSA: Infectious Diseases Society of America
IC: Intensive chemotherapy
MedDRA: Medical Dictionary for Regulatory Activities
MDS: Myelodysplastic syndromes
NCCN: National comprehensive care network
NCI-CTCAE: National Cancer Institute Common Terminology Criteria for Adverse Events
OS: Overall survival
PPI: Proton pump inhibitor
RBC: Red blood cell
RFS: Relapse-free survival
SARS-CoV-2: Severe acute respiratory syndrome coronavirus 2
WBC: White blood cell
