# Supplementary material for: Management of adverse events in patients with acute myeloid leukemia in remission receiving oral azacitidine: experience from the phase 3 randomized QUAZAR AML-001 trial
Source: J Hematol Oncol. 2021 Aug 28;14:133. doi: 10.1186/s13045-021-01142-x (PMC8401338; doi:10.1186/s13045-021-01142-x)
Supplement: Supplementary file 1 — Additional file 1. Additional file of Management of adverse events in patients with acute myeloid leukemia in remission receiving oral azacitidine: experience from the phase 3 randomized QUAZAR AML-001 trial. [file 13045_2021_1142_MOESM1_ESM.docx]

**Supplementary Table 1. Serious adverse events occurring in >2 patients (>1%) in the oral azacitidine arm**

|  | **Oral azacitidine**  **N=236** | **Placebo**  **N=233** |
| --- | --- | --- |
|  | **n (%)** | |
| **Any serious adverse event** | **79 (33)** | **59 (25)** |
| Febrile neutropenia | 16 (7) | 9 (4) |
| Pyrexia | 5 (2) | 1 (< 1) |
| Pneumonia | 9 (4) | 7 (3) |
| Cellulitis | 4 (2) | 1 (< 1) |
| Sepsis | 4 (2) | 5 (2) |
| Atrial fibrillation | 3 (1) | 0 |
| Back pain | 3 (1) | 0 |
| Cholecystitis | 3 (1) | 2 (1) |
| Diarrhea | 3 (1) | 0 |
| Influenza | 3 (1) | 0 |
| A serious AE was any event that resulted in death, was life-threatening, required or prolonged inpatient hospitalization, resulted in significant disability, or led to congenital anomalies or other important medical events. | | |

**Supplementary Table 2. Suggestions for non-pharmacologic management of nausea and vomiting (25)**

| **Practical tips for managing/preventing nausea and vomiting** |
| --- |
| Keep dry crackers near the bed for morning nausea |
| Try chamomile, peppermint, catnip, or ginger tea, and fresh, dried, or candied ginger |
| Sniff a cut lemon |
| Eat salty foods such as pretzels; carry a small packet of salt when leaving home |
| Avoid trigger foods known to cause nausea |
| Avoid strong odors |
| Avoid stomach irritants (e.g., tobacco, aspirin) |
| If there is a pattern to nausea, eat more during periods with less nausea |
| Do not eat and drink at the same time; drink liquids an hour before or after eating |
| Avoid lying down for at least an hour after eating; rest with your head higher than your feet |
| Keep the room temperature cool |
| Avoid eating in a room that is hot, stuffy, or filled with cooking odors |
| **Dietary measures for relieving nausea, vomiting, and diarrhea** |
| Drink clear beverages such as fruit juices, broth, ginger ale, energy drinks, or herbal teas |
| Eat small amounts of food every few hours rather than 2-3 large meals per day |
| Eat slowly and sip beverages slowly |
| Suck on popsicles or frozen fruit juice |
| Try the BRAT diet: bananas, white rice, applesauce, and white bread toast |
| Eat bland, soft foods (e.g., pasta, mashed potatoes, jello) |
| Eat dry foods such as unbuttered toast, saltine crackers, and dry cereal without milk |
| Avoid greasy foods, fried foods, margarine, butter, and oils |
| Avoid spicy foods |
| Avoid dairy products |
| Avoid caffeine (in coffee, tea, soft drinks, chocolate, some pain medications) |
| Avoid alcoholic beverages |
| Avoid acidic foods and juices (eg, citrus fruits, tomatoes) |
| Eat foods high in soluble fiber and avoid foods high in insoluble fiber (eg, nuts) |
| *Adapted from Maceira et al. Ann Palliat Med. 2012;1(2):161-7.* |
